# (*Z*)-3-Methyl-4-[1-(4-methyl­anilino)propyl­idene]-1-phenyl-1*H*-pyrazol-5(4*H*)-one

**DOI:** 10.1107/S1600536813019144

**Published:** 2013-07-17

**Authors:** Naresh Sharma, Komal M. Vyas, R. N. Jadeja, Rajni Kant, Vivek K. Gupta

**Affiliations:** a Post-Graduate Department of Physics & Electronics, University of Jammu, Jammu Tawi 180 006, India; b Department of Chemistry, Faculty of Science, The M.S. University of Baroda, Vadodara 390 002, India

## Abstract

In the title mol­ecule, C_20_H_21_N_3_O, the central pyrazole ring forms dihedral angles of 4.75 (9) and 49.11 (9)°, respectively, with the phenyl and methyl-substituted benzene rings. The dihedral angle between the phenyl and benzene rings is 51.76 (8)°. The amino group and carbonyl O atom are involved in an intra­molecular N—H⋯O hydrogen bond. In the crystal, π–π inter­actions are observed between benzene rings [centroid–centroid seperation = 3.892 (2) Å] and pyrazole rings [centroid–centroid seperation = 3.626 (2) Å], forming chains along [111]. The H atoms of the methyl group on the *p*-tolyl substituent were refined as disordered over two sets of sites in a 0.60 (4):0.40 (4) ratio.

## Related literature

For applications of pyrazole derivatives, see: Wang *et al.* (2005 ▶); Vyas *et al.* (2011 ▶). For general background to Schiff-based pyrazole derivatives, see: Kahwa *et al.* (1986 ▶). For related structures, see: Sharma *et al.* (2012 ▶); Abdel-Aziz *et al.* (2012 ▶).
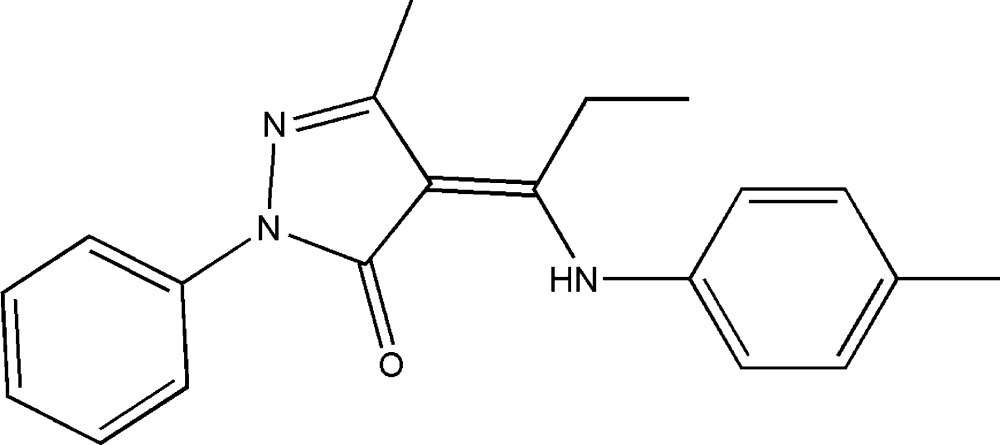


## Experimental

### 

#### Crystal data

C_20_H_21_N_3_O
*M* *_r_* = 319.40
Triclinic, 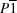

*a* = 8.8092 (3) Å
*b* = 9.8629 (4) Å
*c* = 10.9278 (4) Å
α = 105.633 (4)°
β = 99.971 (3)°
γ = 104.961 (3)°
*V* = 852.75 (5) Å^3^
*Z* = 2
Mo *K*α radiation
μ = 0.08 mm^−1^
*T* = 293 K
0.30 × 0.20 × 0.20 mm

#### Data collection

Oxford Diffraction Xcalibur Sapphire3 diffractometer
Absorption correction: multi-scan (*CrysAlis RED*; Oxford Diffraction, 2010 ▶) *T*_min_ = 0.792, *T*_max_ = 1.000
23723 measured reflections
3341 independent reflections
2067 reflections with *I* > 2σ(*I*)
*R*_int_ = 0.066

#### Refinement

*R*[*F* ^2^ > 2σ(*F* ^2^)] = 0.052
*wR*(*F* ^2^) = 0.130
*S* = 1.01
3341 reflections
225 parameters
H atoms treated by a mixture of independent and constrained refinement
Δρ_max_ = 0.17 e Å^−3^
Δρ_min_ = −0.17 e Å^−3^

### 

Data collection: *CrysAlis PRO* (Oxford Diffraction, 2010 ▶); cell refinement: *CrysAlis PRO*; data reduction: *CrysAlis PRO*; program(s) used to solve structure: *SHELXS97* (Sheldrick, 2008 ▶); program(s) used to refine structure: *SHELXL97* (Sheldrick, 2008 ▶); molecular graphics: *ORTEP-3 for Windows* (Farrugia, 2012 ▶); software used to prepare material for publication: *PLATON* (Spek, 2009 ▶).

## Supplementary Material

Crystal structure: contains datablock(s) I, New_Global_Publ_Block. DOI: 10.1107/S1600536813019144/lh5629sup1.cif

Structure factors: contains datablock(s) I. DOI: 10.1107/S1600536813019144/lh5629Isup2.hkl

Supplementary material file. DOI: 10.1107/S1600536813019144/lh5629Isup3.cml

Additional supplementary materials:  crystallographic information; 3D view; checkCIF report

## Figures and Tables

**Table 1:** Hydrogen-bond geometry (Å, °)

*D*—H⋯*A*	*D*—H	H⋯*A*	*D*⋯*A*	*D*—H⋯*A*
N19—H19⋯O5	0.92 (3)	1.82 (2)	2.656 (2)	151 (2)

**Fig. 1.**
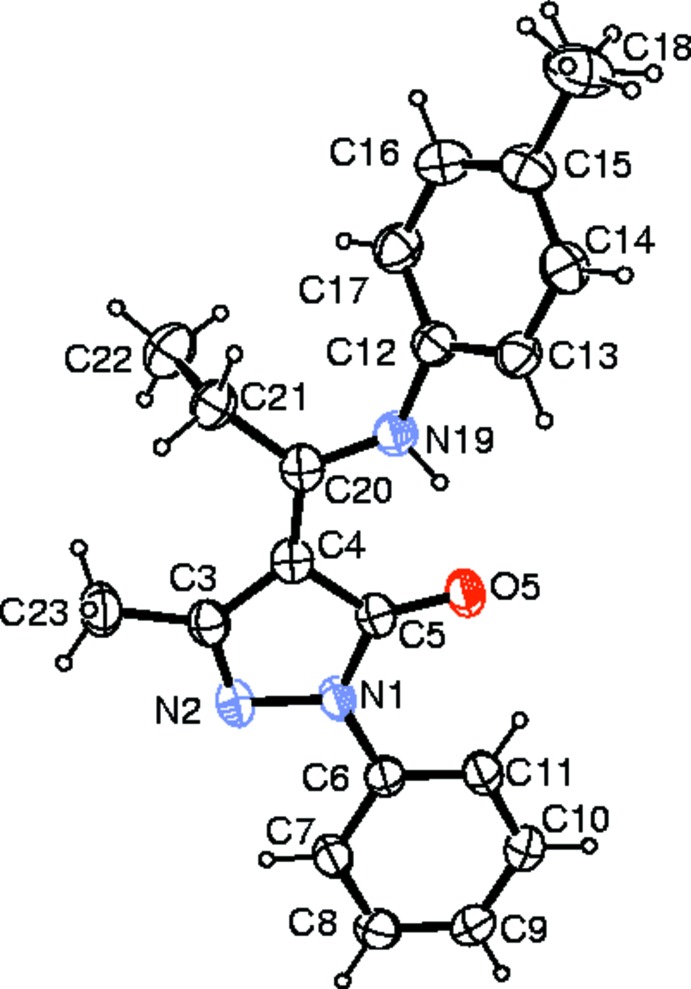
The molecular structure of the title compound. The probabilty ellipsoids are drawn at the 40% probability level. H atoms are shown as small spheres of arbitrary radii.

**Fig. 2.**
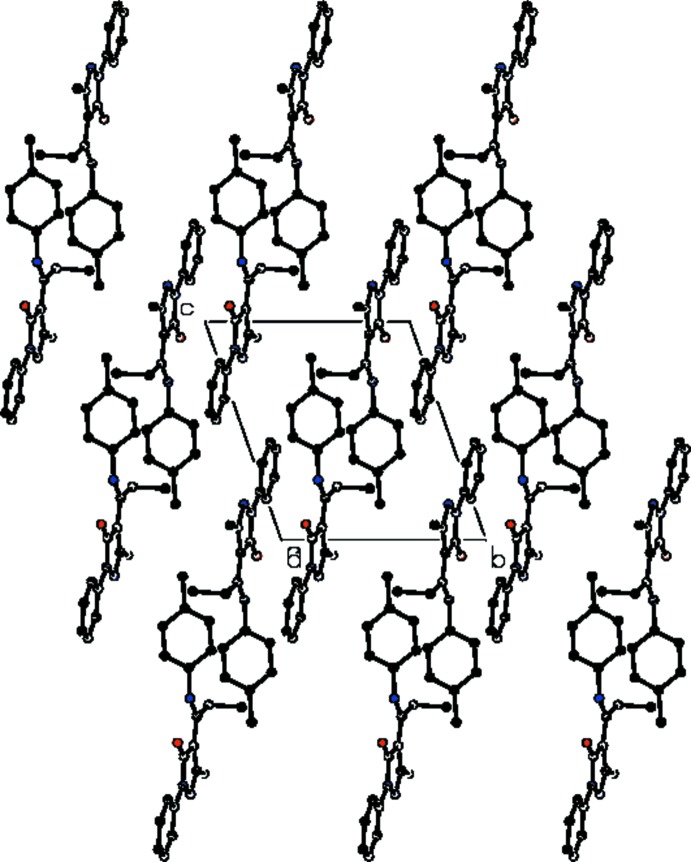
The packing arrangement of molecules viewed along the a axis.

## supplementary crystallographic information

### Comment

Over the past thirty years, extensive chemistry has surrounded the use of Schiff base ligands in inorganic chemistry. Schiff bases of pyrazolone have been playing an important part in the development of coordination chemistry (Kahwa *et al.*, 1986). Consequently, a large number of these species have been reported to be superior reagents in biological, pharmacological, clinical and analytical applications (Wang *et al.*, 2005). As part of an investigation of their crystal structures, which will provide useful information for the coordination properties of Schiff bases functioning as ligands, we report here the synthesis and molecular structure of the title compound. It was prepared as part of our on-going studies of azo dyes with possible medical applications (Vyas *et al.*, 2011). The bond distances in the title compound are comparable to the closely related structures (Abdel-Aziz *et al.*, 2012; Sharma *et al.*, 2012). The central pyrazole (N1/N2/C3/C4/C5) ring makes dihedral angles of 4.75 (9)° and 49.11 (9)°, respectively, with the phenyl (C6-C11) and methyl-substituted benzene (C12-C17) rings. The dihedral angle between the phenyl and benzene rings is 51.76 (8)°. The amino group and the carbonyl oxygen atom are involved in an intramolecular N—H···O hydrogen bond. In the crystal, π···π interactions are observed between the benzene rings [centroid–centroid seperation = 3.892 (2) Å, interplanar spacing = 3.474 Å, centriod shift = 1.75 Å, symmetry code: -*x*,-*y*,-*z*] and pyrazole rings [centroid–centroid seperation = 3.626 (2) Å, interplanar spacing = 3.490 Å, centriod shift = 0.98 Å, symmetry code: 1 - *x*,1 - *y*,1 - *z*] (see Fig. 2).

### Experimental

An equimolar (10 mmol) ethanolic solution (50 ml) of 3-methyl-1-phenyl-4-propionyl-1*H*-pyrazol-5(4*H*)-one and *p*-toluidine was refluxed for 6 h in round bottom flask, whereupon a microcrystalline yellow precipitate appeared. The product was then isolated and recrystallized from ethanol, and then dried in vacuum to give the title compound in 80% yield. Light Yellow single crystals suitable for X-ray analysis were obtained by slow evaporation of an ethanol solution of the title compound.

### Refinement

Atom H19 attached to N19 was located in a difference map and refined isotropically. The remaining H atoms were positioned geometrically and were refined as riding on their parent C atoms, with C—H distances of 0.93–0.97 Å and with *U*_iso_(H) = 1.2*U*_eq_(C), except for the methyl groups where *U*_iso_(H) = 1.5*U*_eq_(C). The H atoms of the methyl group (C18) on the *p*-tolyl substituent were refined as disordered over two sets of sites in a ratio of 0.60 (4):0.40 (4).

### Crystal data

**Table:**

C_20_H_21_N_3_O	*Z* = 2
*M**_r_* = 319.40	*F*(000) = 340
Triclinic, *P*1	*D*_x_ = 1.244 Mg m^−^^3^
Hall symbol: -P 1	Mo *K*α radiation, λ = 0.71073 Å
*a* = 8.8092 (3) Å	Cell parameters from 10378 reflections
*b* = 9.8629 (4) Å	θ = 3.5–29.1°
*c* = 10.9278 (4) Å	µ = 0.08 mm^−^^1^
α = 105.633 (4)°	*T* = 293 K
β = 99.971 (3)°	Block, yellow
γ = 104.961 (3)°	0.30 × 0.20 × 0.20 mm
*V* = 852.75 (5) Å^3^	

### Data collection

**Table:**

Oxford Diffraction Xcalibur Sapphire3 diffractometer	3341 independent reflections
Radiation source: fine-focus sealed tube	2067 reflections with *I* > 2σ(*I*)
Graphite monochromator	*R*_int_ = 0.066
Detector resolution: 16.1049 pixels mm^-1^	θ_max_ = 26.0°, θ_min_ = 3.5°
ω scans	*h* = −10→10
Absorption correction: multi-scan (*CrysAlis RED*; Oxford Diffraction, 2010)	*k* = −12→12
*T*_min_ = 0.792, *T*_max_ = 1.000	*l* = −13→13
23723 measured reflections	

### Refinement

**Table:**

Refinement on *F*^2^	Primary atom site location: structure-invariant direct methods
Least-squares matrix: full	Secondary atom site location: difference Fourier map
*R*[*F*^2^ > 2σ(*F*^2^)] = 0.052	Hydrogen site location: inferred from neighbouring sites
*wR*(*F*^2^) = 0.130	H atoms treated by a mixture of independent and constrained refinement
*S* = 1.01	*w* = 1/[σ^2^(*F*_o_^2^) + (0.0441*P*)^2^ + 0.3811*P*] where *P* = (*F*_o_^2^ + 2*F*_c_^2^)/3
3341 reflections	(Δ/σ)_max_ = 0.001
225 parameters	Δρ_max_ = 0.17 e Å^−^^3^
0 restraints	Δρ_min_ = −0.17 e Å^−^^3^

### Special details

**Table:**

Experimental. *CrysAlis PRO*, Oxford Diffraction Ltd., Version 1.171.34.40 (release 27–08-2010 CrysAlis171. NET) (compiled Aug 27 2010,11:50:40) Empirical absorption correction using spherical harmonics, implemented in SCALE3 ABSPACK scaling algorithm.
Geometry. All e.s.d.'s (except the e.s.d. in the dihedral angle between two l.s. planes) are estimated using the full covariance matrix. The cell e.s.d.'s are taken into account individually in the estimation of e.s.d.'s in distances, angles and torsion angles; correlations between e.s.d.'s in cell parameters are only used when they are defined by crystal symmetry. An approximate (isotropic) treatment of cell e.s.d.'s is used for estimating e.s.d.'s involving l.s. planes.
Refinement. Refinement of *F*^2^ against ALL reflections. The weighted *R*-factor *wR* and goodness of fit *S* are based on *F*^2^, conventional *R*-factors *R* are based on *F*, with *F* set to zero for negative *F*^2^. The threshold expression of *F*^2^ > σ(*F*^2^) is used only for calculating *R*-factors(gt) *etc*. and is not relevant to the choice of reflections for refinement. *R*-factors based on *F*^2^ are statistically about twice as large as those based on *F*, and *R*- factors based on ALL data will be even larger.

### Fractional atomic coordinates and isotropic or equivalent isotropic displacement parameters (Å^2^)

**Table:**

	*x*	*y*	*z*	*U*_iso_*/*U*_eq_	Occ. (<1)
N1	0.6306 (2)	0.5996 (2)	0.37478 (17)	0.0405 (4)	
N2	0.4810 (2)	0.6219 (2)	0.33572 (19)	0.0461 (5)	
C3	0.4497 (3)	0.6918 (2)	0.4430 (2)	0.0432 (5)	
C4	0.5759 (3)	0.7196 (2)	0.5581 (2)	0.0419 (5)	
C5	0.6914 (2)	0.6541 (2)	0.5090 (2)	0.0396 (5)	
O5	0.82042 (18)	0.64696 (18)	0.57192 (14)	0.0524 (4)	
C6	0.6921 (2)	0.5216 (2)	0.2780 (2)	0.0382 (5)	
C7	0.6007 (3)	0.4624 (2)	0.1476 (2)	0.0458 (6)	
H7	0.4989	0.4733	0.1250	0.055*	
C8	0.6609 (3)	0.3874 (3)	0.0520 (2)	0.0542 (7)	
H8	0.5989	0.3479	−0.0351	0.065*	
C9	0.8116 (3)	0.3703 (3)	0.0832 (2)	0.0537 (6)	
H9	0.8524	0.3210	0.0180	0.064*	
C10	0.9002 (3)	0.4274 (3)	0.2122 (2)	0.0540 (6)	
H10	1.0013	0.4149	0.2343	0.065*	
C11	0.8431 (3)	0.5029 (2)	0.3102 (2)	0.0460 (6)	
H11	0.9053	0.5410	0.3971	0.055*	
C12	0.7747 (3)	0.8304 (2)	0.9149 (2)	0.0423 (5)	
C13	0.8209 (3)	0.7326 (3)	0.9708 (2)	0.0456 (6)	
H13	0.8139	0.6388	0.9176	0.055*	
C14	0.8777 (3)	0.7740 (3)	1.1057 (2)	0.0510 (6)	
H14	0.9085	0.7072	1.1424	0.061*	
C15	0.8898 (3)	0.9121 (3)	1.1875 (2)	0.0517 (6)	
C16	0.8441 (3)	1.0085 (3)	1.1292 (2)	0.0558 (7)	
H16	0.8516	1.1025	1.1823	0.067*	
C17	0.7877 (3)	0.9697 (3)	0.9947 (2)	0.0517 (6)	
H17	0.7585	1.0370	0.9580	0.062*	
C18	0.9500 (4)	0.9557 (3)	1.3343 (3)	0.0825 (9)	
H18A	0.9662	1.0595	1.3739	0.124*	0.60 (4)
H18B	1.0513	0.9368	1.3558	0.124*	0.60 (4)
H18C	0.8712	0.8985	1.3671	0.124*	0.60 (4)
H18D	0.9596	0.8703	1.3573	0.124*	0.40 (4)
H18E	0.8745	0.9931	1.3754	0.124*	0.40 (4)
H18F	1.0546	1.0314	1.3641	0.124*	0.40 (4)
N19	0.7247 (2)	0.7827 (2)	0.77489 (19)	0.0468 (5)	
C20	0.5957 (3)	0.7862 (2)	0.6926 (2)	0.0419 (5)	
C21	0.4839 (3)	0.8636 (3)	0.7452 (2)	0.0489 (6)	
H21A	0.4943	0.8702	0.8366	0.059*	
H21B	0.3724	0.8056	0.6963	0.059*	
C22	0.5207 (3)	1.0205 (3)	0.7353 (3)	0.0670 (8)	
H22A	0.4515	1.0688	0.7755	0.101*	
H22B	0.5010	1.0139	0.6444	0.101*	
H22C	0.6325	1.0768	0.7798	0.101*	
C23	0.2922 (3)	0.7244 (3)	0.4309 (3)	0.0591 (7)	
H23A	0.2374	0.6964	0.3396	0.089*	
H23B	0.3132	0.8288	0.4728	0.089*	
H23C	0.2248	0.6690	0.4728	0.089*	
H19	0.778 (3)	0.728 (3)	0.727 (2)	0.063 (8)*	

### Atomic displacement parameters (Å^2^)

**Table:**

	*U*^11^	*U*^22^	*U*^33^	*U*^12^	*U*^13^	*U*^23^
N1	0.0335 (10)	0.0496 (11)	0.0386 (11)	0.0157 (8)	0.0051 (8)	0.0155 (9)
N2	0.0348 (10)	0.0554 (12)	0.0490 (12)	0.0162 (9)	0.0063 (9)	0.0198 (10)
C3	0.0379 (12)	0.0441 (13)	0.0485 (14)	0.0111 (10)	0.0103 (11)	0.0193 (11)
C4	0.0404 (12)	0.0436 (13)	0.0448 (14)	0.0127 (10)	0.0131 (10)	0.0190 (10)
C5	0.0363 (12)	0.0430 (13)	0.0412 (13)	0.0120 (10)	0.0091 (10)	0.0180 (10)
O5	0.0453 (10)	0.0708 (11)	0.0432 (9)	0.0296 (8)	0.0055 (7)	0.0156 (8)
C6	0.0367 (11)	0.0329 (11)	0.0433 (13)	0.0085 (9)	0.0075 (10)	0.0142 (10)
C7	0.0403 (13)	0.0467 (13)	0.0463 (14)	0.0138 (10)	0.0027 (11)	0.0146 (11)
C8	0.0627 (16)	0.0476 (14)	0.0434 (14)	0.0187 (12)	0.0019 (12)	0.0076 (11)
C9	0.0622 (16)	0.0475 (14)	0.0500 (16)	0.0258 (12)	0.0136 (13)	0.0070 (12)
C10	0.0505 (14)	0.0585 (15)	0.0548 (16)	0.0282 (12)	0.0103 (12)	0.0134 (12)
C11	0.0427 (13)	0.0518 (14)	0.0425 (14)	0.0176 (11)	0.0064 (11)	0.0149 (11)
C12	0.0387 (12)	0.0490 (14)	0.0401 (13)	0.0144 (10)	0.0150 (10)	0.0130 (11)
C13	0.0434 (13)	0.0465 (13)	0.0471 (14)	0.0162 (11)	0.0142 (11)	0.0123 (11)
C14	0.0532 (15)	0.0548 (15)	0.0489 (15)	0.0166 (12)	0.0130 (12)	0.0241 (12)
C15	0.0512 (14)	0.0589 (16)	0.0414 (14)	0.0097 (12)	0.0159 (11)	0.0163 (12)
C16	0.0648 (16)	0.0505 (15)	0.0477 (15)	0.0176 (13)	0.0186 (13)	0.0075 (12)
C17	0.0596 (15)	0.0498 (14)	0.0535 (16)	0.0228 (12)	0.0185 (12)	0.0218 (12)
C18	0.104 (3)	0.086 (2)	0.0468 (17)	0.0230 (19)	0.0128 (16)	0.0160 (15)
N19	0.0469 (12)	0.0575 (13)	0.0388 (12)	0.0236 (10)	0.0124 (9)	0.0130 (9)
C20	0.0371 (12)	0.0396 (12)	0.0506 (14)	0.0083 (10)	0.0123 (10)	0.0209 (11)
C21	0.0458 (14)	0.0519 (14)	0.0556 (15)	0.0194 (11)	0.0208 (11)	0.0201 (12)
C22	0.0748 (19)	0.0550 (16)	0.081 (2)	0.0296 (14)	0.0248 (16)	0.0261 (14)
C23	0.0425 (14)	0.0720 (17)	0.0658 (17)	0.0246 (13)	0.0106 (12)	0.0236 (14)

### Geometric parameters (Å, º)

**Table:**

N1—C5	1.372 (3)	C14—C15	1.379 (3)
N1—N2	1.404 (2)	C14—H14	0.9300
N1—C6	1.407 (3)	C15—C16	1.381 (3)
N2—C3	1.304 (3)	C15—C18	1.502 (3)
C3—C4	1.437 (3)	C16—C17	1.379 (3)
C3—C23	1.496 (3)	C16—H16	0.9300
C4—C20	1.398 (3)	C17—H17	0.9300
C4—C5	1.442 (3)	C18—H18A	0.9600
C5—O5	1.252 (2)	C18—H18B	0.9600
C6—C11	1.388 (3)	C18—H18C	0.9600
C6—C7	1.389 (3)	C18—H18D	0.9600
C7—C8	1.378 (3)	C18—H18E	0.9600
C7—H7	0.9300	C18—H18F	0.9600
C8—C9	1.377 (3)	N19—C20	1.335 (3)
C8—H8	0.9300	N19—H19	0.92 (3)
C9—C10	1.371 (3)	C20—C21	1.494 (3)
C9—H9	0.9300	C21—C22	1.534 (3)
C10—C11	1.380 (3)	C21—H21A	0.9700
C10—H10	0.9300	C21—H21B	0.9700
C11—H11	0.9300	C22—H22A	0.9600
C12—C17	1.377 (3)	C22—H22B	0.9600
C12—C13	1.380 (3)	C22—H22C	0.9600
C12—N19	1.425 (3)	C23—H23A	0.9600
C13—C14	1.380 (3)	C23—H23B	0.9600
C13—H13	0.9300	C23—H23C	0.9600
			
C5—N1—N2	111.70 (17)	C12—C17—H17	120.2
C5—N1—C6	129.42 (17)	C16—C17—H17	120.2
N2—N1—C6	118.78 (17)	C15—C18—H18A	109.5
C3—N2—N1	106.56 (17)	C15—C18—H18B	109.5
N2—C3—C4	111.68 (19)	H18A—C18—H18B	109.5
N2—C3—C23	118.1 (2)	C15—C18—H18C	109.5
C4—C3—C23	130.2 (2)	H18A—C18—H18C	109.5
C20—C4—C3	133.0 (2)	H18B—C18—H18C	109.5
C20—C4—C5	122.02 (19)	C15—C18—H18D	109.5
C3—C4—C5	104.92 (19)	H18A—C18—H18D	141.1
O5—C5—N1	125.9 (2)	H18B—C18—H18D	56.3
O5—C5—C4	129.0 (2)	H18C—C18—H18D	56.3
N1—C5—C4	105.10 (17)	C15—C18—H18E	109.5
C11—C6—C7	119.2 (2)	H18A—C18—H18E	56.3
C11—C6—N1	121.19 (19)	H18B—C18—H18E	141.1
C7—C6—N1	119.60 (19)	H18C—C18—H18E	56.3
C8—C7—C6	120.0 (2)	H18D—C18—H18E	109.5
C8—C7—H7	120.0	C15—C18—H18F	109.5
C6—C7—H7	120.0	H18A—C18—H18F	56.3
C9—C8—C7	121.0 (2)	H18B—C18—H18F	56.3
C9—C8—H8	119.5	H18C—C18—H18F	141.1
C7—C8—H8	119.5	H18D—C18—H18F	109.5
C10—C9—C8	118.7 (2)	H18E—C18—H18F	109.5
C10—C9—H9	120.7	C20—N19—C12	131.3 (2)
C8—C9—H9	120.7	C20—N19—H19	108.9 (15)
C9—C10—C11	121.6 (2)	C12—N19—H19	119.2 (15)
C9—C10—H10	119.2	N19—C20—C4	116.9 (2)
C11—C10—H10	119.2	N19—C20—C21	120.1 (2)
C10—C11—C6	119.5 (2)	C4—C20—C21	122.9 (2)
C10—C11—H11	120.3	C20—C21—C22	112.1 (2)
C6—C11—H11	120.3	C20—C21—H21A	109.2
C17—C12—C13	119.5 (2)	C22—C21—H21A	109.2
C17—C12—N19	123.6 (2)	C20—C21—H21B	109.2
C13—C12—N19	116.8 (2)	C22—C21—H21B	109.2
C14—C13—C12	119.9 (2)	H21A—C21—H21B	107.9
C14—C13—H13	120.0	C21—C22—H22A	109.5
C12—C13—H13	120.0	C21—C22—H22B	109.5
C15—C14—C13	121.6 (2)	H22A—C22—H22B	109.5
C15—C14—H14	119.2	C21—C22—H22C	109.5
C13—C14—H14	119.2	H22A—C22—H22C	109.5
C14—C15—C16	117.5 (2)	H22B—C22—H22C	109.5
C14—C15—C18	121.2 (2)	C3—C23—H23A	109.5
C16—C15—C18	121.4 (2)	C3—C23—H23B	109.5
C17—C16—C15	122.0 (2)	H23A—C23—H23B	109.5
C17—C16—H16	119.0	C3—C23—H23C	109.5
C15—C16—H16	119.0	H23A—C23—H23C	109.5
C12—C17—C16	119.6 (2)	H23B—C23—H23C	109.5
			
C5—N1—N2—C3	1.3 (2)	C8—C9—C10—C11	−1.0 (4)
C6—N1—N2—C3	178.00 (18)	C9—C10—C11—C6	0.2 (4)
N1—N2—C3—C4	0.1 (2)	C7—C6—C11—C10	0.6 (3)
N1—N2—C3—C23	−177.35 (19)	N1—C6—C11—C10	−179.1 (2)
N2—C3—C4—C20	−178.3 (2)	C17—C12—C13—C14	0.8 (3)
C23—C3—C4—C20	−1.3 (4)	N19—C12—C13—C14	177.1 (2)
N2—C3—C4—C5	−1.3 (2)	C12—C13—C14—C15	0.0 (4)
C23—C3—C4—C5	175.7 (2)	C13—C14—C15—C16	−0.5 (4)
N2—N1—C5—O5	178.3 (2)	C13—C14—C15—C18	179.4 (2)
C6—N1—C5—O5	2.0 (4)	C14—C15—C16—C17	0.3 (4)
N2—N1—C5—C4	−2.0 (2)	C18—C15—C16—C17	−179.6 (2)
C6—N1—C5—C4	−178.31 (19)	C13—C12—C17—C16	−1.1 (3)
C20—C4—C5—O5	−1.0 (3)	N19—C12—C17—C16	−177.1 (2)
C3—C4—C5—O5	−178.4 (2)	C15—C16—C17—C12	0.5 (4)
C20—C4—C5—N1	179.35 (19)	C17—C12—N19—C20	−51.1 (4)
C3—C4—C5—N1	1.9 (2)	C13—C12—N19—C20	132.8 (3)
C5—N1—C6—C11	−7.2 (3)	C12—N19—C20—C4	−175.5 (2)
N2—N1—C6—C11	176.70 (19)	C12—N19—C20—C21	6.7 (4)
C5—N1—C6—C7	173.0 (2)	C3—C4—C20—N19	174.6 (2)
N2—N1—C6—C7	−3.1 (3)	C5—C4—C20—N19	−2.0 (3)
C11—C6—C7—C8	−0.6 (3)	C3—C4—C20—C21	−7.6 (4)
N1—C6—C7—C8	179.2 (2)	C5—C4—C20—C21	175.8 (2)
C6—C7—C8—C9	−0.2 (4)	N19—C20—C21—C22	98.8 (3)
C7—C8—C9—C10	1.0 (4)	C4—C20—C21—C22	−78.8 (3)

### Hydrogen-bond geometry (Å, º)

**Table:**

*D*—H···*A*	*D*—H	H···*A*	*D*···*A*	*D*—H···*A*
N19—H19···O5	0.92 (3)	1.82 (2)	2.656 (2)	151 (2)
